# Stringent response of *Escherichia coli*: revisiting the bibliome using literature mining

**DOI:** 10.1186/2042-5783-1-14

**Published:** 2011-12-30

**Authors:** Sónia Carneiro, Anália Lourenço, Eugénio C Ferreira, Isabel Rocha

**Affiliations:** 1 Institute for Biotechnology and Bioengineering (IBB), Centre of Biological Engineering, University of Minho, Campus de Gualtar, 4710-057 Braga, Portugal

## Abstract

**Background:**

Understanding the mechanisms responsible for cellular responses depends on the systematic collection and analysis of information on the main biological concepts involved. Indeed, the identification of biologically relevant concepts in free text, namely genes, tRNAs, mRNAs, gene products and small molecules, is crucial to capture the structure and functioning of different responses.

**Results:**

In this work, we review literature reports on the study of the stringent response in *Escherichia coli*. Rather than undertaking the development of a highly specialised literature mining approach, we investigate the suitability of concept recognition and statistical analysis of concept occurrence as means to highlight the concepts that are most likely to be biologically engaged during this response. The co-occurrence analysis of core concepts in this stringent response, i.e. the (p)ppGpp nucleotides with gene products was also inspected and suggest that besides the enzymes RelA and SpoT that control the basal levels of (p)ppGpp nucleotides, many other proteins have a key role in this response. Functional enrichment analysis revealed that basic cellular processes such as metabolism, transcriptional and translational regulation are central, but other stress-associated responses might be elicited during the stringent response. In addition, the identification of less annotated concepts revealed that some (p)ppGpp-induced functional activities are still overlooked in most reviews.

**Conclusions:**

In this paper we applied a literature mining approach that offers a more comprehensive analysis of the stringent response in *E. coli*. The compilation of relevant biological entities to this stress response and the assessment of their functional roles provided a more systematic understanding of this cellular response. Overlooked regulatory entities, such as transcriptional regulators, were found to play a role in this stress response. Moreover, the involvement of other stress-associated concepts demonstrates the complexity of this cellular response.

## Background

Scientific literature represents a valuable source of biological information, in particular on the description of biological entities that we can find in a cellular system and how they are related to each other. To identify references to these entities in texts, here designated as biological concepts, literature mining approaches can be applied. Lately, these approaches have provided for substantial knowledge discovery in diverse biological domains [1-7]. In systems biology, this is of particular interest, since literature-derived evidences can assist in the reconstruction of biochemical and signalling network models. Taking advantage of information retrieval and extraction methodologies it is possible to cover a multitude of biological entities and many other aspects that characterise these complex biological representations. In this work, literature mining was used to get a deep view on the structure of the stringent response in *E. coli*, which was then helpful in the mathematical modelling of this stress response [8].

**Figure 1:**
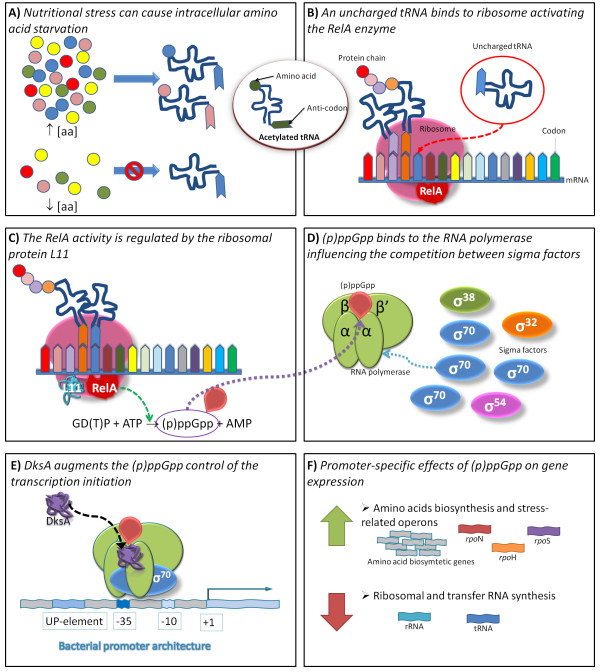
**The (p)ppGpp-mediated stringent response**. (A) Low amino acid concentrations lead to decreased charging of the corresponding tRNAs. (B) The translational machinery depends on the translocation along the mRNA whereby a new acetylated-tRNA is positioned in the ribosome. Whenever an uncharged tRNA binds to the ribosome, the elongation of the polypeptide chain is stalled. (C) The stringent factor RelA is then activated in the presence of the ribosomal protein L11, catalyzing the synthesis of (p)ppGpp nucleotides. (D) These nucleotides bind directly to the RNA polymerase and affect the binding abilities of sigma factors to the core RNA polymerase. (E) The cofactor DksA also binds to the RNA polymerase and augments the (p)ppGpp regulation of the transcription initiation at certain σ70-dependent promoters, functioning both as negative and positive regulators. (F) These regulators change the gene expression: (i) decreasing the transcription activity of genes involved in translational activities; (ii) and increasing the transcription of stress-related operons and genes encoding for enzymes needed for the synthesis and the transport of amino acids.

This stress response has been studied in the last four decades, but many of the cellular mechanisms involved are still unclear [9-14]. Studies have shown that the accumulation of unusual guanosine nucleotides, collectively called (p)ppGpp, is the hallmark of the stringent response of *E. coli *[15-18] (Figure 1). Such accumulation is known to be controlled by the activity of two enzymes, the ribosome-bound RelA enzyme (ppGpp synthetase I) that synthesises (p)ppGpp nucleotides upon the depletion of amino acids [19] and the bifunctional SpoT enzyme (ppGpp synthetase II) that is responsible for maintaining the intracellular levels of (p)ppGpp nucleotides via enzymatic degradation [20]. The (p)ppGpp-mediated response involves the control of the genetic expression by direct interaction of the (p)ppGpp nucleotides with the RNA polymerase (RNAP) [21,22], activating the transcription of genes coding for stress-associated sigma factors and amino acid biosynthesis and inhibiting the transcription of stable RNAs (rRNA and tRNA) [23].

This (p)ppGpp-mediated scenario is quite complex and many fundamental details remain uncertain, such as the mechanisms underlying the activation of transcription by (p)ppGpp [24,25] or the global effects of activating/inhibiting certain stress-related genes [26,27]. To be able to systematically identify and collect information on the main components of the stringent response a semi-automatic literature review process was implemented. We propose the application of a literature mining approach that, besides complementing manual literature review, takes advantage of public database information and ontology assignments to provide for large-scale enrichment and contextualisation of textual evidences. The literature mining approach aimed at (i) corroborating existing knowledge about key players and the processes in which they are involved during the stringent response of *E. coli *and (ii) unveiling knowledge that has been overlooked in the up to date reviews.

## Results

The investigation of the stringent response involved the search for three main biological classes: genetic components (genes, RNA and DNA molecules), gene products (proteins, transcription factors and enzymes) and small molecules. These classes cover for most of the relevant biological concepts involved in this response and each biological concept can be associated with one or more variant names. The annotation of these concepts refers to the mark-up of textual contents that match one of those names.

**Figure 2:**
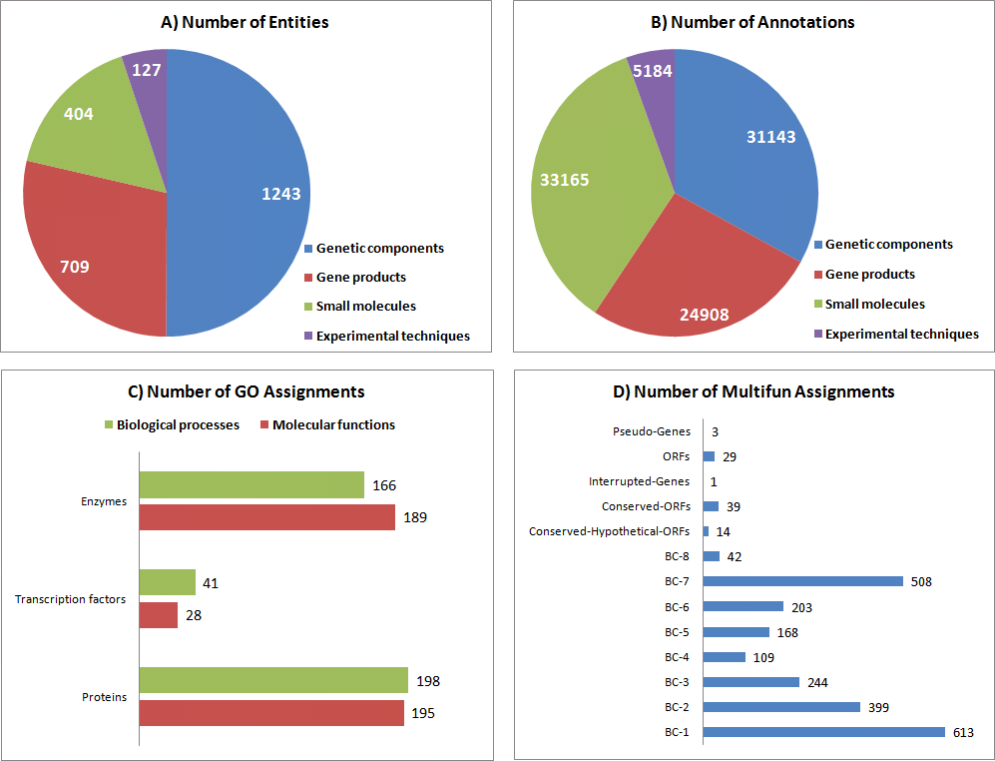
**Corpus annotation contents**. Overview of the extent of biological concepts (A) and concept annotations (B) per class in the corpus. GO assignments (C) for molecular functions and biological processes mapped for each set of gene products (i.e. enzymes, transcription factors and other proteins) and MultiFun gene assignments (D) for different functional roles (BC-1 to Metabolism, BC-2 to Information transfer, BC-3 to Regulation, BC-4 to Transport, BC-5 to Cell processes, BC-6 to Cell structure, BC-7 to Location of gene products and BC-8 to Extrachromosomal origin) were recognized in the corpus.

In the applied literature mining approach, full-text documents related to the stringent response of *E. coli *(published till 2009) were retrieved using NCBI PubMed tools and were further processed to automatically identify and annotate biological concepts in the text. Since we were looking for a wide range of biological concepts, the examination of full-texts was expected to bring much more information. Therefore, the search was limited to documents with link to full-texts. From a total of 251 documents, only 193 full-text documents comprise the corpus in this study, due to the availability of links to full text articles in PubMed and institutional journal subscriptions (see Additional file 1). EcoCyc database [28], a key resource for *E. coli *studies, provided for most of the controlled vocabulary used for the annotation of relevant entities in the documents, namely genes, gene products and small molecules. Proteomics Standards Initiative-Molecular Interactions (PSI-MI) ontology [29] supported the annotation of experimental techniques. After manual curation, i.e. a process to refine and correct annotations, the corpus consisted of 93893 annotations for 2474 biological concepts that were distributed as follows (see Figure 2): genetic components and small molecules accounted for the largest number of annotations (33% and 35% of the overall number of annotations, respectively) and almost half of the biological concepts annotated in full-text documents were classified as genetic components. Additionally, assignments of annotated concepts to MultiFun ontology [30] and Gene Ontology (GO) [31] enabled the identification of biological processes and molecular functions, which were distributed as follows (see Figure 2): enzymes and proteins contributed to most GO assignments; and the MultiFun cellular function categories 'Metabolism'(BC-1) and 'Location of gene products' (BC-7) related to most of the annotated genes. Details are given in Additional file 2.

The analysis of the corpus was based on the assumption that relevant entities could be identified by finding frequent concepts in documents. As such, we measured the frequency of concepts (*freq_ti_*) and the mean (*mean_ti_*) and the standard deviation (*std_ti_*) of the annotations to evaluate their relevance in the discussion throughout the corpus. In other words, the concept frequency (*freq_ti_*) estimated the fraction of documents where a concept was annotated, while the mean (*mean_ti_*) and standard deviation (*std_ti_*) of concept annotations evaluated the distribution of annotations over the corpus. A concept that shows high frequency in the corpus and is annotated more than once per document is likely to have some relevance in the stringent response. To further attest this assumption, we have estimated the variance-to-mean ratio (*VMR_ti_*) that measures the dispersion of annotations for each concept, indicating the existence of document clusters.

We have additionally estimated the frequency of co-annotation (*freq_ti, tj_*) of two concepts, i.e. the number of documents in which concepts *t_i _*and *t_j _*co-occur within the corpus. Co-occurrence analyses has been previously used to establish relationships between biomedical concepts from literature, such as genes and pathways [2,32]. Here we expected to identify pairs of concepts that co-occur frequently in documents, assuming that this frequency is an indication of some sort of relationship between the two concepts and related to the stringent response. Further analyses were performed to evaluate concept annotations per decade, which allowed us to understand the evolving of the topic throughout the years and, in particular, the impact of technology-driven advances. At last, functional enrichment analysis was performed to evaluate which ontology terms were most assigned to annotated concepts. In this analysis we have only considered MultiFun and GO terms related with molecular functions and biological processes, respectively. These results will be detailed in the next subsections.

### Biological concepts

The analysis of the frequency of annotated genetic components (see Table 1) evidenced that entities like the *rel*A gene and the RNA and DNA molecules were annotated in more than 70% of the documents. Though the representativeness of such entities in these documents was considerably high (i.e. high mean of annotation), the annotations were over-dispersed (VMR > 1). For example, the *rel*A gene has a mean of over 22 annotations per document and a VMR of over 33, meaning that some documents present a very high number of annotations, while others rarely mention that gene. This suggests that only a small part of the documents are focused on the discussion of this gene in the stringent response and those are probably the most relevant considering the characterization of its role in this cellular process.

**Table 1:** Annotations of the genetic components in the corpus.

Class	Concept	Number of Annotations	Number of Documents	**% Frequency (Eq. 1)**^**ψ**^	Mean (Eq. 2)	Std (Eq. 3)	VMR (Eq. 4)
Genes	*rel*A	3163	138	71.50	22.92	27.23	33.14
	
	*spo*T	1315	88	45.60	14.94	27.42	52.07
	
	*lac*	354	63	32.64	5.620	19.42	72.20
	
	*lac*Z	534	50	25.91	10.68	17.16	28.90
	
	*thi*	91	47	24.35	1.940	0.050	4.000
	
	*rel*	523	47	24.35	11.13	20.68	36.36
	
	*rec*A	82	39	20.21	2.100	1.810	0.5000
	
	*rps*L	95	36	18.65	2.640	3.530	4.500
	
	*thr*	84	36	18.65	2.330	3.760	4.500
	
	*rps*G	103	34	17.62	3.030	7.250	16.33
	
	*leu*	98	34	17.62	2.880	6.800	18.00
	
	*rpo*S	205	33	17.10	6.210	10.83	16.67
	
	*kan*	308	33	17.10	9.330	16.61	28.44
	
	*gln*V	42	31	16.06	1.350	0.7400	0
	
	*rpo*B	389	30	15.54	12.97	17.60	24.08
	
	*pts*G	240	30	15.54	8.000	21.54	55.13
	
	*trp*	144	25	12.95	5.760	14.73	39.20
	
	*car*A	60	20	10.36	3.000	3.810	3.000
	
	*hsd*R	23	19	9.840	1.210	0.5600	0

DNAs	DNA	1839	137	70.98	13.42	16.31	19.69
	
	plasmid DNA	193	36	18.65	5.360	12.31	28.80
	
	chromosomal DNA	63	24	12.44	2.630	2.440	2.000
	
	cDNA	125	23	11.92	5.430	5.820	5.000

RNAs	RNA	4193	140	72.54	29.95	38.21	49.79
	
	uncharged tRNA	1168	117	60.62	9.980	19.64	40.11
	
	rRNA	1116	97	50.26	11.51	25.97	56.82
	
	a mRNA	999	91	47.15	10.98	19.52	36.10
	
	rrnA	911	87	45.08	10.47	22.51	48.40
	
	stable RNA	430	87	45.08	4.940	8.030	16.00
	
	a charged tRNA	140	43	22.28	3.260	4.200	5.330
	
	rrnB	301	26	13.47	11.58	19.30	32.82
	
	rrn	321	26	13.47	12.35	30.42	75.00
	
	16s-rRNAs	156	25	12.95	6.240	9.090	13.50

Individual genetic components (i.e. genes, DNAs and RNAs) were evaluated considering the number of documents where these entities were annotated and their number of annotations in the corpus. Statistical measurements are detailed in the Methods and Materials section.

^ψ ^A threshold of 10% of the frequency of annotation was set for each genetic component category. However, lists of all annotated entities are provided in Additional file 5.

VMR: variance-to-mean

Std: standard deviation

Similarly, the analysis of gene product annotations (see Table 2) exposed RelA, RNAP and ribosomes as highly annotated entities (present in over than 50% of the documents) with a considerable degree of over-dispersion (VMR > 1). It was interesting to verify that the Fis transcriptional dual regulator, which modulates several cellular processes, such as the transcription of stable RNA (PMID: 2209559; PMID: 9973355)^1 ^[33-35], was highly annotated (with a mean of almost 50 annotations per document), but presented a low frequency (less than 10% of the documents). The extreme value of VMR (over 150) pointed out that some of these documents are devoted to the discussion of this biological entity, which contributed to the large annotation of this concept.

**Table 2:** Annotations of the gene products in the corpus.

Class	Concept	Number of Annotations	Number of Documents	**% Frequency (Eq. 1)**^**ψ**^	Mean (Eq. 2)	Std (Eq. 3)	VMR (Eq. 4)
Proteins	Ribosome	1643	128	66.32	12.84	23.57	44.08
	
	Rel	1021	62	32.12	16.50	36.60	81.00
	
	LacZ	543	53	27.46	10.30	17.44	28.90
	
	Sigma 38 factor	392	42	21.76	9.330	15.40	25.00
	
	Sigma factor	112	35	18.13	3.200	5.870	8.330
	
	UvrD	56	35	18.13	1.600	1.300	1.000
	
	RpoB	252	35	18.13	7.200	11.50	17.29
	
	RecA	99	31	16.06	3.190	4.260	5.330
	
	EF-Tu	223	26	13.47	8.580	17.32	36.13
	
	Der	51	25	12.95	2.040	2.140	2.000
	
	Sigma 70 factor	134	21	10.88	6.380	11.19	20.17

Transcription factors	Fis	888	18	9.330	49.33	86.88	150.9
	
	Fur	56	13	6.740	4.310	9.260	20.25
	
	CRP	279	12	6.220	23.25	36.28	56.35
	
	DnaA	121	11	5.700	11.00	23.00	48.09
	
	H-NS	73	11	5.700	6.640	10.73	16.67
	
	LexA	101	10	5.180	10.10	18.32	32.40
	
	IHF	54	9	4.660	6.000	5.250	4.170

Enzymes	RelA	4138	152	78.76	27.22	31.16	35.59
	
	RNAP	1873	117	60.62	16.01	28.08	49.00
	
	SpoT	1024	60	31.09	17.07	42.19	103.8
	
	EcoRI	215	53	27.46	4.060	4.970	4.000
	
	β-galactosidase	294	47	24.35	6.260	6.550	6.000
	
	BamHI	149	43	22.28	3.470	5.870	8.330
	
	HindIII	114	41	21.24	2.780	2.160	2.000
	
	RNase	109	36	18.65	3.030	4.280	5.330
	
	YbcS	50	23	11.92	2.170	2.620	2.000
	
	Reverse transcriptase	34	21	10.88	1.620	1.050	1.000
	
	tRNA synthetase	54	20	10.36	2.700	2.630	2.000
	
	Endonuclease I	29	20	10.36	1.450	1.400	1.000

Individual gene products (i.e. enzymes, transcription factors and other proteins) were evaluated considering the number of documents where these entities were annotated and their number of annotations in the corpus. Statistical measurements are detailed in the Methods and Materials section.

^ψ ^A threshold of 10% of the frequency of annotation was set for enzymes and other proteins, whereas a threshold of 5% was set for transcription factors. However, lists of all annotated entities are provided in Additional file 6.

VMR: variance-to-mean

Std: standard deviation

The analysis of the annotation of small molecules (see Table 3) revealed that, though almost 83% of the documents discussed the general role of amino acids and nucleotides, the mean of annotation of specific nucleotides and amino acids was quite low (less than 10 annotations per document in most cases). The two exceptions were the nucleotides ppGpp and (p)ppGpp (the collective reference for ppGpp and pppGpp). A high frequency (75% and 37%, respectively) and mean of annotation (29 and 43, respectively) confirm that these nucleotides are central in the stringent response in *E. coli*. Indeed, during amino acid starvation (p)ppGpp nucleotides coordinate several cellular activities by influencing gene expression.

**Table 3:** Annotations of the small molecules in the corpus.

Concept	Number of Annotations	Number of Documents	**% Frequency of annotation (Eq. 1)**^**ψ**^	Mean of annotation (Eq. 2)	Std (Eq. 3)	VMR (Eq. 4)
Amino acids	1557	160	82.90	9.730	13.83	18.78

Nucleotides	1230	145	75.13	8.480	9.290	10.13

ppGpp	4159	145	75.13	28.68	31.00	34.32

β-D-glucose	792	123	63.73	6.440	10.63	16.67

Pi	662	113	58.55	5.860	12.60	28.80

Guanosine	407	112	58.03	3.630	3.540	3.000

ATP	587	100	51.81	5.870	7.410	9.800

GTP	748	91	47.15	8.220	13.85	21.13

AMP	598	90	46.63	6.640	10.09	16.67

PPi	447	87	45.08	5.140	5.180	5.000

H_2_O	210	83	43.01	2.530	2.430	2.000

Tris	261	82	42.49	3.180	2.800	1.330

Carbon	288	80	41.45	3.600	4.850	5.330

Chloramphenicol	435	77	39.90	5.650	8.250	12.80

pppGpp	632	74	38.34	8.540	13.61	21.13

(p)ppGpp	3127	72	37.31	43.43	56.00	72.93

NaCl	189	67	34.72	2.820	2.790	2.000

L-lactate	413	65	33.68	6.350	20.84	66.67

Glycerol	145	65	33.68	2.230	1.850	0.5000

Ethanol	189	65	33.68	2.910	4.400	8.000

Na^+^	145	63	32.64	2.300	2.100	2.000

Ampicillin	321	62	32.12	5.180	12.74	28.80

EDTA	142	60	31.09	2.370	1.680	0.5000

L-methionine	248	59	30.57	4.200	6.670	9.000

L-histidine	183	59	30.57	3.100	5.410	8.330

L-valine	396	57	29.53	6.950	11.90	20.17

Formate	136	57	29.53	2.390	2.360	2.000

Individual small molecules were evaluated considering the number of documents where these entities were annotated and the number of annotations in the corpus. Statistical measurements are detailed in the Methods and Materials section.

^ψ ^A threshold of 30% of the frequency of annotation was set for compounds. However, lists of all annotated entities are provided in Additional file 7.

VMR: variance-to-mean

Std: standard deviation

**Figure 3:**
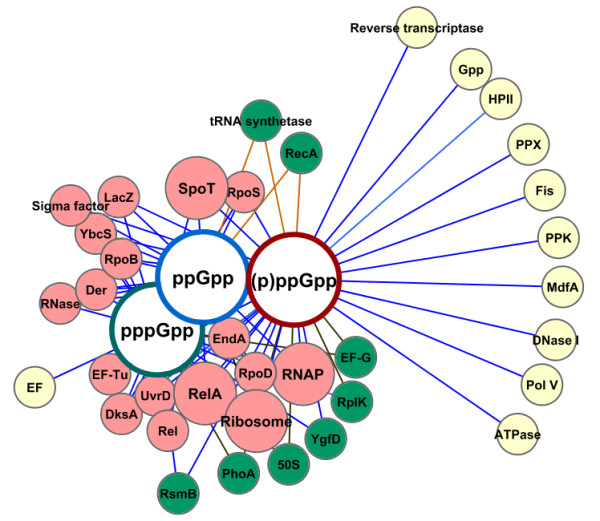
**Proteins co-annotated with ppGpp, pppGpp and the collective (p)ppGpp entities**. The nodes represent proteins with frequency of co-annotation higher than 10%. Highly co-annotated proteins are represented by nodes with a larger size (frequencies of co-annotation greater than 50%). Pink nodes represent the proteins that were co-annotated with the three entities, while green and yellow nodes indicate the proteins that were co-annotated with only two and one of the nucleotides, respectively.

Taking these results into account, the frequency of co-annotation of these nucleotides with gene products was evaluated. As mentioned above, it can be admitted that the co-occurrence of two concepts (*t_i _*and *t_j_*) in the same body text might indicate a relationship between them (even if indirect). Thus, to find key players in the stringent response that are possibly affected by the accumulation of (p)ppGpp nucleotides, the frequency of co-annotation of concepts for gene products and each of these nucleotides was estimated. As shown in Figure 3 and Additional file 3, (p)ppGpp nucleotides were found to be considerably co-annotated with highly representative proteins, namely: the RelA and SpoT enzymes that control the basal levels of the nucleotides (in 93% and 67% of the (p)ppGpp-annotated documents, respectively); ribosomes that are affected by the nucleotides activity (in approximately 79% of the (p)ppGpp-annotated documents); RNAP (in approximately 64% of the (p)ppGpp-annotated documents); and the RpoS, the alternative sigma factor σ^38 ^that acts as the master regulator of the general stress response (in approximately 40% of the (p)ppGpp-mentioning documents) (PMID: 9326588) [35]. Some proteins were co-annotated with only one or two of the concepts. For instance, the Gpp enzyme that converts pppGpp into ppGpp (PMID: 8531889; PMID: 6130093) [36,37] was essentially co-annotated with the pppGpp concept. In turn, the RecA protein, which catalyses DNA strand exchange reactions (PMID: 17590232) [38], and the tRNA synthetase were co-annotated with (p)ppGpp and ppGpp with a frequency higher than 10%, whereas other proteins were mainly co-annotated with (p)ppGpp and pppGpp: the elongation factor (EF) G, known to facilitate the translocation of the ribosome along the mRNA molecules (PMID: 8531889) [36]; the RplK (or 50S ribosomal subunit protein L11) that was reported to be essential when the 30S ribosomal initiation complex joins to the 50S ribosomal subunit and in the EF-G-dependent GTPase activity (PMID: 17095013; PMID: 12419222) [39,40]; and the enzyme PhoA known to be involved in the acquisition and transport of phosphate (PMID: 9555903) [41].

Additionally, results pointed out potentially interesting associations with less represented proteins (see Additional file 3), such as: the Fur transcriptional activator that controls the transcription of genes involved in iron homeostasis (PMID: 15853883) [42]; the HN-S transcriptional dual regulator that is capable of condensing and supercoiling DNA (PMID: 10966109) [43]; the DnaA protein implicated in the chromosomal replication initiation (PMID:1690706) [44]; the DinJ-YafQ complex involved in the inhibition of protein synthesis and growth (PMID:12123445) [12] and the MazE antitoxin of the MazF-MazE toxin-antitoxin system involved in translation inhibition processes (PMID:12123445) [12]. In general, most co-annotated entities correspond to gene products that have regulatory functions in the gene transcription process, such as transcriptional factors, or that are components of the translational apparatus.

### Examining less-reported entities

**Figure 4:**
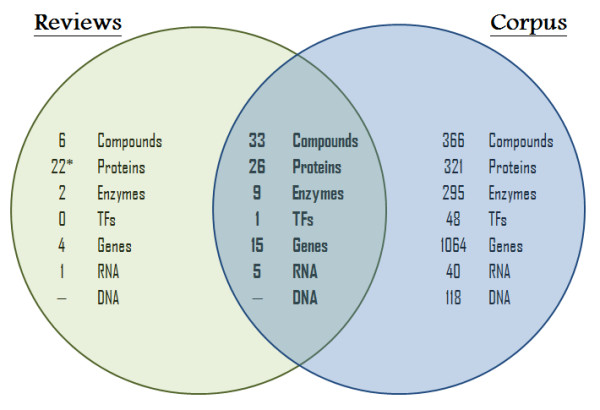
**Venn diagram comparing annotations from corpus and selected reviews**. This diagram indicates the number of biological concepts per class that represent the corpus and from the latest reviews considered to be relevant to this subject. The intersecting zone gives the number of biological concepts that were simultaneously reported in the two set of documents.

In the present corpus, most of the biological entities identified as major participants in the *E. coli *stringent response, were also extensively cited in recent reviews [9,11,17,24,25] (see Additional file 4). These reviews were selected based on their relevance in terms of information specifically collected for the stringent response in *E. coli *and their number of citations. As illustrated in Figure 4, biological concepts considered to be key components in those reviews were also evidenced by the semi-automatic information extraction approach.

However, when examining the extent of annotated concepts from the selected reviews and the corpus, it was evident that many biological entities have been disregarded or less reported in the reviews. Biological entities, such as transcriptional factors and other gene products like stress-related proteins, were not described in the selected reviews. Indeed, often the role of some biological entities that are directly (or indirectly) associated with the stringent response, is missing in most literature revisions. To illustrate this, we compiled information on the participation of some less-reported biological entities in *E. coli *stringent response by validating their role. These results can be found in Table 4.

**Table 4:** Some examples of less-reported entities (namely in recent reviews), which are relevant in the *E. coli *stringent response.

Biological entities	Freq (%)	Details	References
DnaJ - chaperone with DnaK	3.11	Chaperone protein that assists the DnaJ/DnaK/GrpE system of *E. coli*. The overproduction of ppGpp has shown to induce the accumulation of these chaperones.	[68]

ClpB chaperone	1.55	ClpB, together with the DnaJ/DnaK/GrpE chaperone system, is able to resolubilize aggregated proteins.	[69]

GroEL-GroES chaperonin complex	0.52	GroEL and GroES are both induced by heat and when ppGpp is overproduced in *E. coli*.	[68]

RuvB - branch migration of Holliday structures; repair helicase	1.55	Component of the RuvABC enzymatic complex that promotes the rescue of stalled (often formed by ppGpp) or broken DNA replication forks in *E. coli*.	[70]

CsrA - carbon storage regulator	1.04	Regulator of carbohydrate metabolism, which activates UvrY, responsible for the transcription of *csr*B that, in turn, inhibits the CsrA activity.	[71]

*uvr*Y	0.52	Encodes the UvrY protein that has been shown to be the cognate response regulator for the BarA sensor protein. This regulator participates in controlling several genes involved in the DNA repair system (e.g. CsrA) and carbon metabolism.	[72]

*cst*A	0.52	Gene encoding the CstA peptide transporter, which expression is induced by carbon starvation and requires the CRP-cAMP transcriptional regulator. The CstA translation is regulated by the CsrA that occludes ribosome binding to the *cst*A mRNA.	[73]

CspD - DNA replication inhibitor	0.52	CspD is a toxin that appears to inhibit the DNA replication. ppGpp is one of the positive factors for the expression of *csp*D.	[74]

FabH - β-ketoacyl-ACP synthase III	0.52	A key enzyme in the initiation of fatty acids biosynthesis that is stringently regulated by ppGpp.	[75]

FadR transcriptional dual regulator	1.55	Regulates the fatty acid biosynthesis and fatty acid degradation at the level of transcription. ppGpp has been shown to be also involved in the regulation of these pathways	[75]

NtrC-Phosphorylated transcriptional dual regulator	1.04	Regulatory protein involved in the assimilation of nitrogen and in slow growth caused by N-limited condition. It was reported that ppGpp levels increase upon nitrogen starvation.	[76]

*dps*	2.59	Gene encoding the Dps protein that is highly abundant in the stationary-phase and is required for the starvation responses. It was found to be regulated by ppGpp and RpoS.	[77,78]

*psi*F	2.07	Gene induced during phosphate starvation that has been associated with the accumulation of ppGpp.	[41]

*chp*R	2.07	Encodes the MazE antitoxin, a component of the MazE-MazF system that causes a "programmed cell death" in response to stresses, including starvation. Genes *maz*E and *maz*F are located in the *E. coli rel *operon and are regulated by ppGpp.	[79]

*maz*G	0.52	Encodes the MazG nucleoside pyrophosphohydrolase that limits the detrimental effects of the MazF toxin under nutritional stress conditions. Overexpression of *maz*G inhibits cell growth and negatively affects accumulation of ppGpp.	[80]

The recognition of these proteins in the corpus was invaluable, allowing to uncover various stress-responsive proteins, such as chaperones (e.g. DnaJ, ClpB or the GroEL-GroES chaperonin complex) and toxin-antitoxin systems (e.g. protein encoded by *chp*R) that are normally associated with other stress responses. The description of such entities as participants in the stringent response discloses a more insightful overview of the complexity of these entangled cellular processes. For example, the identification of entities related to certain metabolic pathways, like the fatty acids biosynthesis (e.g. FabH and FabR), or DNA processes, like DNA replication (e.g. CspD) and DNA repair (e.g. *uvr*Y), can expand the characterization of stringently regulated activities that have not been evident in previous reviews.

### Evolution of technology *versus *knowledge

From the previous results it is clear that studies on the stringent response were essentially focused on the characterizations of the catalytic activities of the enzymes RelA and SpoT and their role in the control of (p)ppGpp accumulation. Though these processes are central in the stringent response, the global effects of the (p)ppGpp-dependent response are of primordial importance. The influence on transcription and translation processes and the triggering of other stress responses have raised interest from researchers.

**Figure 5:**
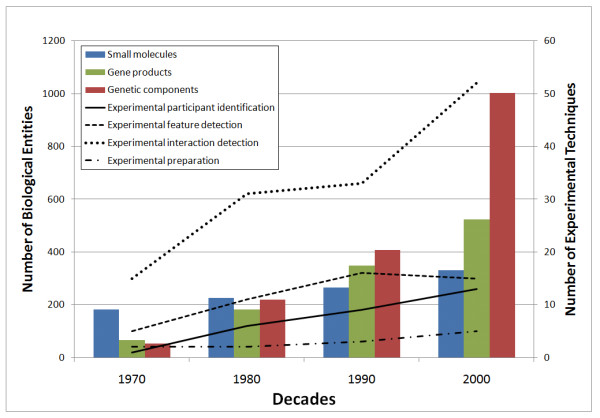
**Comparison of the expansion of knowledge to the applied experimental techniques**. Bars represent the number of biological entities (left Y axis) found for the three major biological classes, i.e., genetic components (genes, RNAs and DNAs), gene products (proteins, transcription factors and enzymes) and small molecules. Lines plot the number of experimental techniques (right Y axis) associated to the annotated PSI-MI classes.

As such, the investigation of many other biological entities and their roles during the stringent response has driven research to the systems-wide understanding of this complex regulatory networks [12]. Only with the development of more sophisticated techniques, like peptide mass fingerprinting (MI:0082) or chromatin immunoprecipitation arrays (MI:0225), it was possible to investigate the complexity of biological systems in a high throughput manner. The development of experimental techniques was a key point in this transition and it is expected to intersect points of turnover on the study of the stringent response. We could confirm that with the availability of more experimental techniques, the extent of biological concepts annotated in our corpus increased. By comparing the number of annotations of biological concepts (genetic components, gene products and small molecules) and experimental techniques (grouped into major PSI-MI classes) per decade (Figure 5) it is possible to verify the progression of knowledge related with the stringent response in *E. coli*.

The analysis evidenced that the repertoire of experimental techniques has been growing significantly and the study is ever more dedicated to the investigation of genetic components. In particular, results showed the use of an ever-growing number of experimental interaction detection methods (MI:0045) and a considerable number of experimental participant identification (MI:0661) and experimental feature detection (MI:0659) methods. The analysis of annotated experimental techniques in the corpus (Table 5) evidenced that the chromatography technology (MI:0091), experimental feature detection (MI:0657), genetic interference (MI:0254) and primer specific polymerase chain reaction (PCR) (MI:0088) techniques were annotated in more than 40% of the documents. Most techniques were referred roughly two times per document, but primer-specific PCR (MI:0088) and array technology (MI:0008) presented a considerable mean of annotation (with over 10 and 8 annotations per document, respectively) and high VMR values (22.5 and 6.13, respectively), which indicated that these techniques were essentially discussed in a given set of documents.

**Table 5:** PSI-MI assignments to annotated experimental techniques.

Techniques			Statistics over the corpus				Frequency per Decade			
**PSI-MI Class**	**PSI-MI id**	**PSI-MI name**	**Freq**	**Mean**	**Std**	**VMR**	**1970**	**1980**	**1990**	**2000**

**MI:0659 experimental feature detection**	MI:0659	experimental feature detection*	55%	2.44	2.76	2	64%	65%	67%	67%
	
	MI:0833	autoradiography	25%	1.65	1.4	1	29%	35%	22%	22%
	
	MI:0113	western blot	21%	4.95	3.38	2.25	-	13%	18%	44%
	
	MI:0074	mutation analysis	20%	3.34	3.24	3	14%	20%	27%	32%
	
	MI:0114	x-ray crystallography	4%	1.63	1.46	1	-	-	2%	8%
	
	MI:0811	insertion analysis	4%	1.14	0.38	0	-	-	7%	4%

**MI:0045 experimental interaction detection**	MI:0091	chromatography technology	50%	4.23	4.14	4	100%	85%	55%	64%
	
	MI:0254	genetic interference	42%	2.68	2.35	2	-	28%	69%	64%
	
	MI:0807	comigration in gel electrophoresis	37%	2.51	2.13	2	21%	48%	51%	49%
	
	MI:0045	experimental interaction detection*	36%	3.1	3.12	3	14%	45%	47%	41%
	
	MI:0808	comigration in sds page	27%	2.02	1.61	0.5	7%	23%	33%	29%
	
	MI:0099	scintillation proximity assay	24%	1.94	1.65	1	64%	35%	20%	15%
	
	MI:0051	fluorescence technology	16%	1.84	1.8	1	7%	20%	5%	22%
	
	MI:0071	molecular sieving	15%	2.25	3.19	4.5	29%	13%	15%	13%
	
	MI:0217	phosphorylation reaction	13%	2.72	3.74	4.5	7%	8%	16%	14%
	
	MI:0415	enzymatic study	12%	1.96	2.11	4	7%	8%	11%	19%
	
	MI:0008	array technology	10%	8.47	7.47	6.13	-	-	-	22%
	
	MI:0928	filter trap assay	9%	2.24	2.47	2	36%	13%	4%	6%
	
	MI:0004	affinity chromatography technology	8%	2.33	2.44	2	-	5%	5%	13%
	
	MI:0428	imaging technique	7%	1.57	1.41	1	-	8%	4%	11%
	
	MI:0047	far western blotting	6%	1.5	0.82	0	-	3%	7%	8%
	
	MI:0435	protease assay	6%	3.92	4.01	5.33	-	5%	5%	8%
	
	MI:0017	classical fluorescence spectroscopy	6%	1.08	0.29	0	-	-	5%	11%
	
	MI:0089	protein array	6%	1.64	1.62	1	-	3%	2%	11%
	
	MI:0029	cosedimentation through density gradient	5%	5.22	3.94	1.8	43%	10%	-	2%
	
	MI:0040	electron microscopy	4%	2.57	1.31	0.5	-	10%	-	4%
	
	MI:0676	tandem affinity purification	3%	3.8	5.18	8.33	-	-	-	7%
	
	MI:0054	fluorescence-activated cell sorting	3%	5.8	4.6	3.2	-	-	4%	4%
	
	MI:0413	electrophoretic mobility shift assay	3%	1.6	0.77	0	-	-	5%	2%
	
	MI:0012	bioluminescence resonance energy transfer	2%	6.25	5.68	4.17	-	-	4%	2%
	
	MI:0018	two hybrid	2%	2	1.41	0.5	-	-	-	7%
	
	MI:0053	fluorescence polarization spectroscopy	2%	5	2.94	0.8	-	3%	-	2%
	
	MI:0397	two hybrid array	2%	2	1.41	0.5	-	-	2%	2%
	
	MI:0227	reverse phase chromatography	2%	3.25	2.87	1.33	-	-	5%	1%
	
	MI:0226	ion exchange chromatography	1%	1	0	0	-	-	-	1%
	
	MI:0031	protein cross-linking with a bifunctional reagent	1%	7	4	2.29	-	3%	-	1%
	
	MI:0052	fluorescence correlation spectroscopy	1%	1	0	0	-	-	-	1%
	
	MI:0416	fluorescence microscopy	1%	2.5	0.71	0	-	-	-	2%
	
	MI:0016	circular dichroism	1%	1.5	0.71	0	-	-	-	2%
	
	MI:0225	chromatin immunoprecipitation array	1%	1	0	0	-	-	-	1%
	
	MI:0872	atomic force microscopy	1%	1	0	0	-	-	-	1%
	
	MI:0049	filter binding	1%	1	0	0	-	3%	2%	-
	
	MI:0426	light microscopy	1%	1	0	0	-	-	-	2%

**MI:0661 experimental participant identification**	MI:0088	primer specific pcr	40%	10.38	15.87	22.5	-	8%	29%	95%
	
	MI:0080	partial dna sequence identification by hybridization	27%	3.75	3.47	3	14%	30%	29%	26%
	
	MI:0078	nucleotide sequence identification	20%	1.77	1.26	1	-	15%	25%	22%
	
	MI:0103	southern blot	14%	3.04	2.05	1.33	-	8%	15%	19%
	
	MI:0929	nothern blot	8%	5.56	4.8	3.2	-	3%	11%	11%
	
	MI:0421	identification by antibody	6%	1.82	1.51	1	-	8%	5%	6%
	
	MI:0427	identification by mass spectrometry	5%	1.67	0.94	0	-	-	4%	8%
	
	MI:0082	peptide massfingerprinting	2%	1.5	0.71	0	-	-	-	5%
	
	MI:0093	protein sequence identification	1%	1	0	0	-	-	2%	
	
	MI:0411	enzyme linked immunosorbent assay	1%	4	2	1	-	-	2%	1%

**MI:0346 experimental preparation**	MI:0714	nucleic acid transduction	26%	2.22	2.82	2	14%	15%	31%	29%
	
	MI:0715	nucleic acid conjugation	6%	1.73	1.41	1	7%	3%	5%	7%
	
	MI:0308	electroporation	5%	1.89	1.56	1	-	-	2%	9%
	
	MI:0343	cdna library	3%	1	0	0	-	-	-	6%

**MI:0190 interaction type**	MI:0194	cleavage reaction	1%	1	0	0	-	3%	-	-

**MI:0116 feature type**	MI:0373	dye label	5%	1.2	0.45	0	7%	8%	5%	4%

Experimental techniques were evaluated considering the number of documents where these concepts were annotated and the number of annotations in the corpus. Moreover, the frequency of annotation of experimental techniques was also estimated for documents published in the four decades (from 1970 to 2009). Statistical measures are detailed in the Methods and Materials section.

* These PSI-MI general classes were used to identify techniques that did not map into any particular technique within the class.

A detailed look into the frequency of concept annotation per decade points out that some of the techniques used in early studies have a reduced application today and highlights the increasing influence of high-throughput technologies in recent studies. For instance, experimental interaction detection methods (MI:0045), such as the scintillation proximity assay (MI:0099), the molecular sieving (MI:0071), the filter trap assays (MI:0928) and the cosedimentation through density gradient (MI:0029) were mostly annotated in documents from the first decade (1970-1980), whereas the comigration in gel electrophoresis (MI:0807) and enzymatic studies (MI:0415) were increasingly reported in documents throughout the decades.

### Functional enrichment

Recent developments in the functional annotation of genomes using biological ontologies provided the means to contextualise literature mining outputs. The automatic mapping of annotated concepts to ontology terms was facilitated by EcoCyc database information that supports the assignment of MultiFun and GO ontology terms to genes and gene products. MultiFun ontology classifies gene products according to their cellular function, namely: metabolism, information transfer, regulation, transport, cell processes, cell structure, location, extra-chromosomal origin, DNA site, and cryptic gene. In turn, GO embraces three separate ontologies: cellular components, i.e. the parts of a cell or its extracellular environment; molecular functions, i.e. the basic activities of a gene product at the molecular level, such as binding or catalysis; and biological processes, i.e. the set of molecular events related to the integrated functioning of cells, tissues, organs or organisms.

Here, we have analysed the assignments for MultiFun cellular functions and GO biological processes (Table 6 and Table 7). The aim was to find statistically overrepresented ontology terms related with cellular functions and biological processes within the set of annotated concepts. Seemingly to what is proposed in some bioinformatics tools [45,46], where gene expression datasets are analysed to find statistically over- or under-represented terms, we have estimated the frequency of ontology terms assigned to annotated concepts in the corpus (regarding both gene and gene products). For calculating this frequency of assignment, the fraction of documents in the corpus that included those ontology terms was also considered. For example, if a GO term is assigned to one or more concepts that altogether were annotated in 80% of the documents, than the frequency of assignment of that ontology term in the corpus is 80%.

**Table 6:** MultiFun cellular function assignments.

MultiFun Concepts	Frequency of Ontology Annotation	Brief Description	Genes			Gene Products	
			
			Name	Frequency of Assignment	Frequency of Annotation	Name	Frequency of Annotation
BC-1.7.33 Nucleotide and nucleoside conversions	76%	The chemical reactions involved in the central carbon metabolism by which a nucleobase, nucleoside or nucleotide is converted from another nucleobase, nucleoside or nucleotide.	*rel*A	68%	72%	RelA	79%
			
			*spo*T	28%	46%	SpoT	31%

BC-3.1.3.4 Proteases, cleavage of compounds	55%	Proteins that hydrolysates a peptide bond or bonds within a protein during posttranscriptional regulatory processes.	*spo*T	91%	46%	SpoT	31%

BC-2.2.2 Transcription related functions	51%	The information transfer related functions involved in the synthesis of RNA on a template of DNA.	*fis*	22%	6%	Fis	9%
			
			*rpo*B	17%	16%	RpoB	18%

BC-2.3.2 Translation	48%	The cellular metabolic process by which a protein is formed, using the sequence of a mature mRNA molecule to specify the sequence of amino acids in a polypeptide chain.	*dks*A	23%	3%	DksA	8%
			
			*rpl*K	17%	6%	RplK	7%
			
			*rps*G	12%	18%	RpsG	NA
			
			*rps*L	11%	19%	RpsL	3%

BC-5.5.3 Starvation	47%	A state or activity of a cell or an organism as a result to the adaptation to starvation.	*spo*T	85%	46%	SpoT	31%
			
			*dks*A	12%	3%	DksA	8%

BC-1.1.1 Carbon compounds	46%	The metabolic reactions by which living organisms utilises carbon compounds.	*lac*Z	49%	26%	LacZ	28%
			
			*pts*G	22%	16%	PtsG	1%

BC-2.3.8 Ribosomal proteins	44%	Proteins that associate to form a ribosome involved in genetic information transfer in cells.	*rpl*K	24%	6%	RplK	7%
			
			*rps*G	18%	18%	RpsG	NA
			
			*rps*L	17%	19%	RpsL	3%

BC-3.1.2.3 Repressor	40%	Any transcription regulator that prevents or downregulates transcription.	*fis*	41%	6%	Fis	9%

BC-3.1.2.2 Activator	32%	Any transcription regulator that induces or upregulates transcription.	*fis*	45%	6%	Fis	9%

MultiFun terms were assigned to annotated concepts associated with genes. A threshold of 30% of documents was considered for ontology assignment and a threshold of 10% was used to point out the genes that most contributed to such assignment.

NA: corresponds to non-annotated gene products in the corpus.

**Table 7:** GO biological processes assignments.

Gene Ontology concepts	Frequency of Ontology Annotation	Brief Description	Gene Products			Coding Genes	
			
			Name	Frequency of Assignment	Frequency of Annotation	Name	Frequency of Annotation
GO:0008152 Metabolic process	89%	The chemical reactions and pathways, including anabolism and catabolism, by which living organisms transform chemical substances.	RelA	80%	79%	*rel*A	72%
			
			LacZ	10%	24%	*lac*Z	26%

GO:0015949 Nucleobase, nucleoside and nucleotide interconversion	80%	The chemical reactions and pathways by which a nucleobase, nucleoside or nucleotide is synthesized from another nucleobase, nucleoside or nucleotide.	RelA	80%	79%	*rel*A	72%
			
			SpoT	20%	31%	*spo*T	46%

GO:0015969 Guanosine tetraphosphate metabolic process	80%	The chemical reactions and pathways involving guanine tetraphosphate (5'-ppGpp-3'), a derivative of guanine riboside with four phosphates.	RelA	80%	79%	*rel*A	72%
			
			SpoT	20%	31%	*spo*T	46%

GO:0006350 Transcription	56%	The synthesis of either RNA on a template of DNA or DNA on a template of RNA.	RpoS	16%	22%	*rpo*S	17%
			
			CRP	12%	6%	*crp*	4%
			
			RpoB	10%	18%	*rpo*B	16%
			
			Mfd	10%	2%	*mfd*	2%

GO:0006355 Regulation of transcription, DNA-dependent	52%	Any process that modulates the frequency, rate or extent of DNA-dependent transcription.	RpoS	20%	22%	*rpo*S	17%
			
			CRP	14%	6%	*crp*	4%
			
			Mfd	12%	2%	*mfd*	2%

GO:0006412 Translation	40%	The cellular metabolic process by which a protein is formed, using the sequence of a mature mRNA molecule to specify the sequence of amino acids in a polypeptide chain.	RplK	28%	7%	*rpl*K	6%
			
			DksA	28%	8%	*dks*A	3%
			
			EF-Tu	13%	14%	*tuf*B	3%

GO:0006950 Response to stress	39%	A change in state or activity of a cell or an organism as a result of a disturbance in cellular homeostasis, usually, but not necessarily, exogenous.	RecA	20%	16%	*rec*A	20%
			
			RelB	16%	4%	*rel*B	3%
			
			NusA	10%	5%	*nus*A	4%

GO:0042594 Response to starvation	39%	A change in state or activity of a cell or an organism as a result of a starvation stimulus, deprivation of nourishment.	SpoT	67%	31%	*spo*T	46%
			
			DksA	30%	8%	*dks*A	3%

GO:0006970 Response to osmotic stress	38%	A change in state or activity of a cell or an organism as a result of a stimulus indicating an increase or decrease in the concentration of solutes outside the organism or cell.	RpoS	59%	22%	*rpo*S	17%
			
			EF-Tu	34%	14%	*tuf*B	3%

GO:0005975 Carbohydrate metabolic process	36%	The chemical reactions and pathways involving carbohydrates, any of a group of organic compounds based of the general formula Cx(H2O)y.	LacZ	94%	24%	*lac*Z	26%

GO:0006974 Response to DNA damage stimulus	36%	A change in state or activity of a cell or an organism as a result of a stimulus indicating damage to its DNA from environmental insults or errors during metabolism.	Mfd	28%	2%	*mfd*	2%
			
			RecA	11%	16%	*rec*A	20%
			
			RecG	11%	3%	*rec*G	NA

GO:0006281 DNA repair	36%	The process of restoring DNA after damage that include direct reversal, base excision repair, nucleotide excision repair, photoreactivation, bypass, double-strand break repair pathway, and mismatch repair pathway.	Mfd	28%	2%	*mfd*	2%
			
			RecA	11%	16%	*rec*A	20%
			
			RecG	11%	3%	*rec*G	NA

GO terms were assigned to annotated concepts associated with gene products annotations. A threshold of 35% of documents was considered for ontology assignment and a threshold of 10% was used to point out the gene products that most contributed to such assignment.

NA: corresponds to non-annotated genes in the corpus.

We have also evaluated the contribution of concept annotations to the assignment of an ontology term. The frequency of annotation of a given term (A) by a given concept (B) was therefore estimated based on the ratio of the number of annotations of the concept (B) by the number of times that ontology term (A) was assigned by any concept associated to that term (A). Since one concept can be associated to several ontology terms, it can be considered that the under- or over-representation of an ontology term can depend on the number of annotations of the assigned concepts. In this perspective, concepts that were highly annotated in the corpus were considered the most relevant for the assignment of an ontology term. In summary, we estimate which are the most important cellular processes involved in the stringent response and also the key biological concepts involved in these processes.

The analysis of MultiFun cellular function assignments (Table 6) evidenced gene functions related to central metabolism processes, post-transcriptional processes and transcription-related functions (covered by over 50% of the documents). The most assigned MultiFun cellular functions, namely metabolic functions related to nucleotide and nucleoside conversions (BC-1.7.33) and proteolytic cleavage of compounds (BC-3.1.3.4), derived from the highly annotated *rel*A and *spo*T genes. The *lac*Z gene, another highly annotated gene (26% of the documents), that encodes the β-galactosidase enzyme responsible for the hydrolysis of β-galactosides into monosaccharides, contributed significantly (almost 50% of assignments) to the annotation of cellular functions implicated in the metabolism of carbon compounds (BC-1.1.1). However, in this particular case it should be acquainted that the occurrence of this gene in documents is frequently associated with molecular assays using *lac*Z as a reporter gene. Therefore, the relevance of this gene to the stringent response might be questionable.

The gene *fis *that encodes the Fis transcriptional dual regulator and the gene *rpo*B coding for the β subunit of the RNAP, contributed the most to the annotation of transcriptional related functions (BC-2.2.2). Similarly, genes like *dks*A that encodes the DksA protein, *rpl*K that encodes the 50S ribosomal subunit L11, and *rps*G and *rps*L coding for the 30S ribosomal subunits S7 and S12, respectively, contributed the most to the annotation of translation related processes (BC-2.3.2). By looking into the frequencies, it was verified that there is a discrepancy of annotation between the genes contributing to enriched ontology terms and the corresponding gene products. Therefore, the use of the MultiFun ontology not only pointed out relevant gene function assignments, but also disclosed the participation of several gene products that, even being less reported in documents, were highlighted by functional association. Some examples are the 30S ribosomal subunit protein S12 and the 30S ribosomal subunit protein S7 encoded by *rps*L and *rps*G, respectively.

On the other hand, the analysis of GO biological process assignments (Table 7) highlighted metabolic and genetic information transfer processes as the most frequently assigned (i.e., over 50% of the documents have annotated concepts that were assigned to these ontology terms). Besides the general term "metabolic process" having the highest frequency (89% of the documents), two particular terms associated with metabolic processes: the "nucleobase, nucleoside and nucleotide interconversion process" (GO:0015949) and the "guanosine tetraphosphate metabolic process" (GO:0015969), had high frequencies (around 80% of the documents) as well. The gene product that contributed the most to the annotation of terms related with metabolic processes was the RelA enzyme, with over 80% of the assignments. Regarding ontology terms related with genetic information transfer, "transcription" (GO:0006350), "DNA-dependent transcription regulation" (GO:0006355) and "translation" (GO:0006412) were the most represented processes (56%, 52% and 40% of the documents, respectively). The RpoS or the alternative sigma factor σ^38 ^that acts as the master regulator of the general stress response, the CRP transcriptional dual regulator, known to participate in the transcriptional regulation of genes involved in the degradation of non-glucose carbon sources and the Mfd protein, found to be responsible for ATP-dependent removal of stalled RNAPs from DNA, contributed similarly to the annotation of transcription and DNA-dependent transcription regulation processes, ranging between 10% and 20% of the assignments. Translation process assignments were derived from the RplK (or 50S ribosomal subunit protein L11) and the DksA proteins, with 28% of the assignments each, and the Elongation Factor Tu (EF-Tu), which mediates the entry of the aminoacyl tRNA into the ribosome, with 13% of the assignments.

We have paid particular attention to stress-specific ontology terms like the "response to stress" (GO: 0006950), the "response to starvation" (GO: 0042594), the "response to osmotic stress" (GO: 0006970) and the "response to DNA damage stimulus" (GO:0006974) that were assigned in almost 40% of the documents. The GO term "response to stress" was mostly assigned by the RecA regulatory protein, the RelB transcriptional repressor and the transcription antitermination protein NusA (frequencies of assignment of 20%, 16% and 10%, respectively). Regarding the GO term "response to starvation", SpoT enzyme detached from other contributing gene products (almost 70% of the assignments). The σ^38 ^factor and the EF-Tu protein were the main contributors to the assignment of the GO term "response to osmotic stress" (59% and 34% of the assignments, respectively), while the assignment of "response to DNA damage stimulus" was mainly due to the annotation of proteins like Mfd, RecA and RecG (28%, 11% and 11% of the assignments, respectively). The process of restoring DNA after damage, associated with the GO term "DNA repair" (GO:0006281), was also assigned by the aforementioned annotated entities.

Since results evidenced considerable assignment of stress-related processes, it was considered interesting to explore in detail the functional annotations of gene products related to *E. coli *stress responses (Table 8). A decade-by-decade analysis was performed to evaluate the extent of documents that study entities associated with these functional annotations. As shown, the response to starvation (GO:0042594) was mostly evidenced in the last decade, being assigned in almost 70% of the documents of this decade. The response to DNA damage stimulus (GO:0006974) and osmotic stress (GO:0006970) were also considerably assigned in the last decade (50% of the documents). On the contrary, the defense response to bacterium (GO:0042742) was less assigned in the documents from the last two decades (less than 10% of the documents) and the stringent response (GO:0015968) was poorly assigned in the last decade, probably because GO only associates this biological process to the 50S ribosomal subunit protein L11, which only recently has been studied in the context of this stress.

**Table 8:** Assignment of GO concepts related to stress responses.

			Frequency of Ontology Annotation			
**GO Identifier**	**GO Concept**	**GO Description**	**1970**	**1980**	**1990**	**2000**

GO:0042594	Response to starvation	A change in state or activity of a cell or an organism as a result of a starvation stimulus, deprivation of nourishment.	-	15%	28%	68%

GO:0006974	Response to DNA damage stimulus	A change in state or activity of a cell or an organism as a result of a stimulus indicating damage to its DNA.	7%	26%	31%	50%

GO:0006970	Response to osmotic stress	A change in state or activity of a cell as a result of an increase or decrease in the concentration of solutes outside the cell.	21%	28%	35%	50%

GO:0006950	Response to stress	A change in state or activity of a cell or an organism as a result of a disturbance in organismal or cellular homeostasis.	-	31%	46%	46%

GO:0006979	Response to oxidative stress	A change in state or activity of a cell or an organism as a result of oxidative stress.	-	3%	20%	45%

GO:0009432	SOS response	An error-prone process for repairing damaged microbial DNA.	-	23%	30%	45%

GO:0046677	Response to antibiotic	A change in state or activity of a cell or an organism as a result of an antibiotic stimulus.	-	23%	30%	15%

GO:0042493	Response to drug	A change in state or activity of a cell or an organism as a result of a drug stimulus.	-	15%	35%	11%

GO:0009266	Response to temperature stimulus	A change in state or activity of a cell or an organism as a result of a temperature stimulus.	-	-	9%	11%

GO:0042742	Defense response to bacterium	Reactions triggered in response to the presence of a bacterium that act to protect the cell or organism.	14%	26%	9%	8%

GO:0015968	Stringent response	A specific global change in the metabolism of a bacterial cell as a result of starvation.	-	13%	7%	6%

GO:0009408	Response to heat	A change in state or activity of a cell or an organism as a result of a heat stimulus.	-	3%	13%	5%

GO:0009409	Response to cold	A change in state or activity of a cell or an organism as a result of a cold stimulus.	-	-	-	3%

GO:0009636	Response to toxin	A change in state or activity of a cell or an organism as a result of a toxin stimulus.	-	-	-	3%

GO:0009267	Cellular response to starvation	A change in state or activity of a cell as a result of deprivation of nourishment.	-	-	-	1%

GO:0046688	Response to copper ion	A change in state or activity of a cell or an organism as a result of a copper ion stimulus.	-	-	-	1%

GO:0009269	Response to desiccation	A change in state or activity of a cell or an organism as a result of a desiccation stimulus.	-	-	4%	-

GO:0031427	Response to methotrexate	A change in state or activity of a cell or an organism as a result of a methotrexate stimulus.	-	-	2%	-

The frequency of annotation of stress response-related concepts was estimated for documents published in the four decades analysed (from 1970 to 2009).

## Discussion

The aim of this work was to use literature mining to complement manual curation in the revision, systematisation and interpretation of current knowledge on the stringent response of *E. coli*. Literature mining was expected to help on the identification of important biological players and their molecular functions. The controlled vocabulary extracted from the EcoCyc repository (i.e. concepts that identify biological entities like genetic components, gene products and small molecules) and ontology terms from GO, MultiFun and PSI-MI ontologies were expected to support large-scale information processing and biological contextualisation.

The application of literature mining approaches has been tested in different biological fields [3,4,6,47-50], but it is known that the quality of the information extracted has to be ensured by manual curation. At present, manual curation can extract more detailed information from literature than it is possible by mining approaches, and more accurately define the participants and their roles. However, to achieve a broad coverage, both approaches can efficiently complement each other. As such, we propose a semi-automated approach to revise, systematise and interpret the current knowledge on the stringent response of *E. coli*, based on specific controlled vocabulary for the identification of biological entities involved in this process and complemented with manual curation. Results suggested that: (i) automatic literature retrieval is able to provide documents of interest whereas controlled vocabulary from publicly available databases can support the identification of relevant entities; (ii) ontology assignments enable entity contextualisation into cellular functions and biological processes, delivering a more comprehensive and biologically meaningful scenario; and (iii) statistical analysis identifies biological entities of interest and facilitates document indexing for additional manual curation. Ultimately, the literature mining approach presented clues on entities and associations of interest and suggested which documents in the corpus should be further inspected for details on given entities or processes.

The analysis performed to the final set of annotated concepts in the corpus, evidenced the (p)ppGpp nucleotides as some of the most annotated biological entities: the ppGpp nucleotide was annotated in 75% of the documents, and the broad term (p)ppGpp exhibited the highest average of annotations per document (Table 3). The extensive number of documents supporting these annotations evidenced that the role of (p)ppGpp nucleotides in the stringent response has been extensively studied. Corpus analysis disclosed that the *rel*A gene product was also extensively studied. Indeed, over 70% of the documents addressed the activity of the *rel*A gene and its product RelA. This enzyme was first associated to the synthesis of ppGpp in 1970 (PMID: 4315151) [51]. It is described that during amino acid deprivation the accumulation of this nucleotide increases above basal levels. Later, in 1980, the ppGpp level was found to be controlled by the SpoT enzyme via GTP hydrolysis activity (PMID: 6159345) [52]. However both the *spo*T gene and the SpoT enzyme have been annotated in only roughly 30% of the documents. In part, because RelA was the first enzyme discovered to be involved in the stringent response, but mostly because it is the first biological entity to respond to the amino acid starvation. Accordingly, "nucleobase, nucleoside and nucleotide interconversion" emerged as one of the most assigned ontology terms in the corpus, mainly due to the high frequency of assignment allocated to the RelA protein (80%). Transcriptional and translational processes were also highlighted by the analysis. The acknowledgment that (p)ppGpp nucleotides manipulate gene expression, so that gene products with important roles in the starvation survival are favoured at the expense of those required for growth and proliferation, has been widely reported (PMID:12123445; PMID:10809680) [12,53]. *In vitro *studies demonstrated that (p)ppGpp bind directly to the RNAP, affecting the transcription of many genes (PMID:4553835) [54]. Also, studies hypothesised that the configuration of the RNAP is altered, decreasing the affinity of the housekeeping sigma factor (i.e. σ^70^) to RNAP and thus, allowing other sigma factors to compete and influence promoter selectivity (PMID:12023304) [55]. As covered by the corpus analysis, besides RNAP (annotated in over 60% of the documents), four of the existing sigma factors in *E. coli *were also annotated: the σ^38 ^that acts as the master regulator of the general stress response (annotated in 22% of the documents); the σ^70 ^that is the primary sigma factor during exponential growth (annotated in 11% of the documents); the σ^54 ^that controls the expression of nitrogen-related genes (annotated in 4% of the documents); and the σ^32 ^that controls the heat shock response during log-phase growth (annotated in 3% of the documents). Although the regulation of transcription initiation is not yet fully understood, current knowledge suggests that these four sigma factors may interact with the RNAP during stringent control.

Regarding transcription-related ontology terms, the concepts that contributed the most to these assignments were: the β subunit of the RNAP (RpoB) to which (p)ppGpp nucleotides bind (PMID:9501189) [56]; the CRP transcriptional dual regulator that is activated in response to starvation conditions (PMID:10966109) [43]; the Fis transcriptional dual regulator, whose gene promoter is inhibited during the transcription initiation by the (p)ppGpp-bound RNAP (PMID:2209559; PMID:9973355) [33,34]; and the Mfd protein that releases the arrested RNAP-DNA complexes after (p)ppGpp nucleotides induce the transcription elongation pausing, protecting genome integrity during transient stress conditions (PMID:7968917) [57]. It is known that (p)ppGpp nucleotides not only modulate the RNAP activity, either by reducing the expression of genes like *fis *(which in turn modulates the expression of the *crp *gene) or increasing the expression of the σ^38 ^gene, but also mediate the inhibition of the RNAP replication-elongation, which afterwards requires the Mfd protein to remove the stalled RNAPs (PMID:16039593; PMID:7968917) [57,58]. Although most studies have focused on the influence of the (p)ppGpp nucleotides on the mechanisms that regulate transcription initiation activities, their regulatory effects on the elongation of DNA transcription are also important. The combined control of the DNA transcription initiation and elongation are central to a prompter cellular response to nutritional starvation [11], which has been highlighted in the present study by the presence of associated concepts in the analysis.

Similarly, (p)ppGpp also influence certain translation-related processes. Studies showed that (p)ppGpp inhibits translation by repressing the expression of ribosomal proteins and also potentially inhibiting the activity of the particular proteins (PMID:7021151; PMID:11673421; PMID:6358217) [59-61]. Corpus analysis evidenced the annotation of ribosomal proteins, such as the 50S ribosomal subunit protein L11 and the 30S ribosomal subunit proteins S7 and S12, as well as the EF-Tu and the non-ribosomal DksA protein. The 50S ribosomal subunit protein L11 has been indirectly implicated in the feedback inhibition of (p)ppGpp, because ribosomes lacking this protein are unable to stimulate the synthesis of these nucleotides (PMID:11673421; PMID:17095013) [39,61]. The involvement of the DksA protein in translation processes was inferred through the inspection of functional assignments. As reported (PMID:16824105) [62], DksA regulates the posttranscriptional stability of σ^38 ^factor, which increases dramatically when (p)ppGpp levels are high. Although these are the main (p)ppGpp interactions at the translational level, the impact of these nucleotides in the translation apparatus was further analysed based on the frequency of co-annotation of gene products with (p)ppGpp nucleotides that unveiled additional participants at this level. As a result, it was possible to perceive the relevance of specific translation GTPases known to be inhibited by (p)ppGpp nucleotides, namely: the Der protein that stabilises the 50S ribosomal subunit and the EF-G that facilitates the translocation of the ribosome along the mRNA molecules (PMID:8531889) [36].

Apart from identifying and contextualising numerous biological participants in the stringent response, the proposed analysis (in particular, the analysis of GO functional assignments) suggested that some of the biological entities involved in other stress responses may also participate in the stringent response. Responses to starvation, DNA damage and osmotic, oxidative and SOS stresses are some examples of stress responses that were also evidenced in the analysis of the corpus (over 30% of the documents). Yet, it was striking to notice that the stringent response concept was barely assigned, probably because few biological entities are currently associated with this GO term. In fact, the 50S ribosomal subunit protein L11 was the only entity in this corpus associated with that term. Nevertheless, several biological entities that interplay in different responses to stress were identified in the corpus, suggesting the overlap between stress responses. Although the relationship between these entities and the stringent response in this study is merely hypothetical, it was verified that their participation in the stringent response has been experimentally tested. For example: the link between the stringent response and the response to osmotic and oxidative stresses is likely to be via the involvement of the σ^38 ^factor and the EF-Tu protein; the response to DNA damage stimulus was assigned to the RecA, RecG and Mfd proteins that were verified in literature to intervene in the early dissociation of the elongation complex stalled by ppGpp [58]; and finally, the RecA regulator and the UvrABC nucleotide excision repair complex have been implicated in the DNA repair process and SOS response [63].

With the extensive list of biological players retrieved from the corpus, it was possible to recognize and investigate most of the (p)ppGpp induced cellular processes. The major participants in the stringent response were highlighted by their frequency of annotation and their representativeness in the corpus. The (p)ppGpp nucleotides and the RelA and SpoT enzymes that control (p)ppGpp basal levels, along with the RNAP, were pointed as the most significant entities in the corpus. Nonetheless, corpus analysis also revealed the involvement of entities that have been disregarded or less reported in most recent revisions [9,11,17,24,25]. In most cases, this is due to the fact that the reviews are not focused on the detailed description of the molecular mechanisms involved in the stringent response. They reflect the current state of knowledge, including the different levels of cellular processes that are triggered during this stress response, but do not specify which biological entities are involved in these processes. However, researchers often need to compile this information, not only for experimental purposes, but also for computational modelling or to better understand the complexity of the response. Hence, in this study, the stringent response was associated with a large set of biological entities. We have validated the role of many of those entities by inspecting directly the literature used to build the corpus, therefore also validating the usefulness of the methodology developed. However, further validation would be necessary to have a broad description of the stringent response considering the large variety of biological entities directly or indirectly affected by the (p)ppGpp within specific metabolic, transcriptional and translational processes. The systematization of information on the stringent response of *E. coli *is therefore the greatest benefit from the methodology described in this work.

Besides collecting and organizing information into functional classes, we were able to analyse the advances accomplished in the investigation of the stringent response in *E. coli*. Comparing the annotations of biological concepts and concepts associated with experimental techniques (based on the PSI-MI ontology terms) we followed the evolution of knowledge being reported along four decades. Technological developments have promoted the discovery of many new entities and have clarified their roles in the stringent response. At the early stage of the study of the stringent response, some traditional experimental techniques were considered decisive in the identification of the main metabolic participants (see Figure 5 and Table 5), such as the (p)ppGpp nucleotides that have been investigated since the 70s. Yet, in the last decades, research efforts have been focused on the newest molecular biology techniques, namely high-throughput detection methods. In particular, techniques based on array technology have addressed the rapid screening of biological entities as well as molecular interactions (PMID: 18039766; PMID: 17233676) [64,65]. DNA microarrays have been used to inspect the genome-wide transcriptional profiles of *E. coli *(PMID: 18039766) [64]. This technology has also provided information on transcriptional regulation, determining negatively controlled promoters (typically involved in cell growth and DNA replication) and positively controlled promoters (the amino acid biosynthesis, the transcription factors, and/or alternative sigma factor genes). Although the reconstruction of the transcriptional regulatory structure of the stringent response is far from complete, these recent advances have brought a closer view of the pleiotropic nature of the response.

Finally, results showed that it is possible to scale-up conventional manual curation coping with the ever-increasing publication rate and, at the same time, provide automatic means of identifying and contextualising participants of interest. Beyond the accomplishments of the approach on this particular study, its extension to the analysis of other stress responses and/or organisms is fairly easy and interesting. However, although in the case of bacterial systems this might be simple, where naming conventions are straightforward and mostly followed by the community, in the case of other organisms, like Drosophila or plant systems, there will be more limitations. Adaptation to other scenarios implicates the compilation of sets of related documents and specialized controlled vocabularies that can be more elaborate and complex.

## Methods

### Semi-automatic information extraction approach

**Figure 6:**
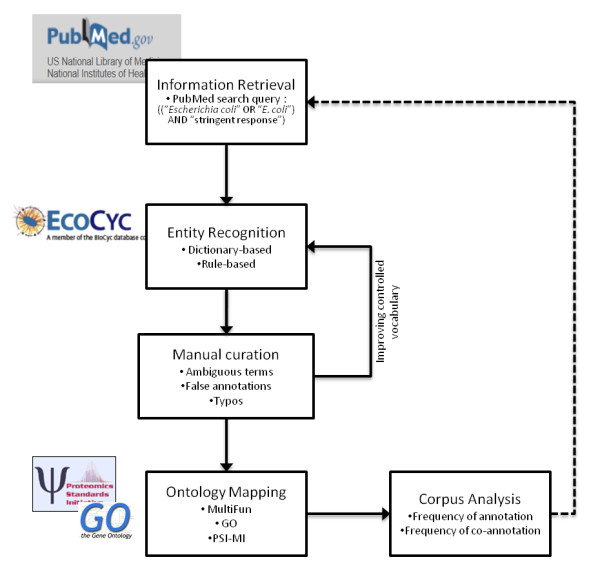
**Semi-automatic information extraction approach**. The first step encompasses the retrieval of relevant documents that are then processed to recognize biological concepts. In the following step, a manual curation procedure is undertaken to ensure the quality of the final corpus. Ontological terms are further mapped to enable functional enrichment analysis. The corpus analysis enables the identification of key players or significant information by an incremental curation that can further deliver information for retrieving new relevant documents.

The semi-automatic information extraction approach designed to review existing literature on the stringent response of *E. coli*, integrated the following procedures: automatic document retrieval and entity recognition processes, manual curation and corpus analysis (see Figure 6).

The documents to be analysed were compiled through PubMed keyword-based searches in January 2010, using the terms ("*Escherichia coli*" OR "*E. coli*") and some variants of the term "stringent response" as reference. The process of document retrieval was limited to full-texts and it retrieved a total of 251 documents, from which only 193 full-text documents were used due to the availability of links to full text articles in PubMed and institutional journal subscriptions. The list of documents is provided in the Additional file 1.

The automatic identification of biological concepts in those documents was done in a subtask so-called entity recognition that seeks to locate and classify concepts in texts. These concepts correspond to names or textual descriptions that are listed in a structure called controlled vocabulary that was built using information from the EcoCyc database [28], a key resource for *E. coli *studies, and includes biological concepts used for the recognition of genetic components (genes, RNA and DNA molecules), gene products (i.e. proteins, including transcription factors and enzymes) and small molecules (or metabolites). Additionally, a hand-crafted dictionary supported the recognition of experimental techniques and their association to PSI-MI ontology terms. For each recognized concept in documents we have estimated the number of annotations, i.e. the number of times that that concept appears in the body texts (including all name variants associated with that biological concept).

@Note [66], a workbench for Biomedical Text Mining supported the entity recognition process. Its regular expression module enabled the identification of genes and proteins that adhere to standard gene and protein naming conventions for *E. coli *(e.g. three lower case letters followed by a fourth letter in upper case or a term consisting of four digits preceded by character 'b' are candidates for gene names) while the dictionary-based recognition module used the terminology extracted from EcoCyc and PSI-MI ontology.

The annotated corpus was stored into XML files for further analysis. The manual curation process consisted on reviewing concept annotations, i.e., the text markups (XML tags) for recognised entities, to ensure the corpus quality and consistency. Errors of the automated recognition process such as the annotation of false entities (e.g. the word 'cap', a name variant for the CRP transcriptional factor, was wrongly annotated or words like 'release' or 'crease' were annotated as enzymes based on common enzyme suffix 'ase'), homonyms (e.g. the same term 'elongation factor Tu' to designate two different polypeptides 'TufA' and 'TufB') and PDF-to-text format conversion typos (e.g. '4azaleucine' and '9galactosidase' were corrected to '4-azaleucine' and 'β-galactosidase', respectively) were manually curated.

### Controlled vocabulary

EcoCyc database (version 13.0, released in March 2009) provided for most of the controlled vocabulary. It supported the automatic identification of genetic components, gene products and small molecules as follows: common names and extensive name variants (synonyms) were used in the recognition of concept names in the texts; name variants were normalised by associating the corresponding database record identifier, which unequivocally identifies the concept, to the annotation; and database assignments to Gene Ontology (GO) [31] and MultiFun [30] terms enabled the mapping of annotated concepts, like gene and gene products, to the associated molecular functions and biological processes. These ontology assignments were straightforward since all entries in the EcoCyc dictionary keep the corresponding database assignments to these ontologies. Additional vocabulary was extracted from Proteomics Standards Initiative-Molecular Interactions (PSI-MI) ontology for the annotation of experimental techniques.

### Analysis methodology

The number of annotations of a concept, the number of documents that contributed for those annotations and the number of documents composing the corpus constituted the baseline of the statistical metrics used in the analyses. Let *D *be the set of documents in the corpus and *T *be the set of annotated concepts in *D*. For every *t_i _*∈ *T*, the frequency, the mean, the standard deviation of annotation, and the variance-to-the mean ratio (or coefficient of dispersion) were computed as described in Table 9.

**Table 9:** Annotation statistics used in the analysis.

Frequency	$freq_{t_{i}}\;=\frac{D_{t_{i}}}{D}=\frac{\#docs_{t_{i}}}{\#docs}$	(Eq. 1)
**Mean**	$mean_{t_{i}}\;=\mu_{{}_{t_{i}}}\;=\frac{\#annots_{t_{i}}}{\#docs_{t_{i}}}$	(Eq. 2)

**Standard deviation**	$std_{t_{i}}=\sigma_{t_{i}}=\sqrt{1\;/\;\#docs_{t_{i}}\overset{\#docs_{t_{i}}}{\underset{j=1}{\sum }}{\left(\#annots_{t_{i},doc_{j}}-\mu_{t_{i}}\right)}^{2}}$	(Eq. 3)

**Variance-to-mean**	$VMR_{t_{i}}=\frac{{\sigma_{t_{i}}}^{2}}{\mu_{t_{i}}}$	(Eq. 4)

**Co-annotation**	$freq_{t_{i},t_{j}}=\frac{D_{t_{i}\cap t_{j}}}{D_{t_{i}}}=\frac{\#docs_{t_{i}\cap t_{j}}}{\#docs_{t_{i}}}$	(Eq. 5)

The frequency of annotated concepts, *freq_ti _*(Eq.1), estimates the fraction of documents in *D *that refers the concept *t_i_*. In turn, the mean, *μ_ti _*(Eq.2), and the standard deviation, *σ_ti _*(Eq.3), weight the number of annotations of a concept, *#annots_ti_*, in the documents in *D *that include that concept, *docs_ti_*, and measure the average or dispersion of the annotations, respectively. The mean indicates the representativeness of the concept in the subset *docs_ti _*whereas the standard deviation indicates the variability of annotations in the subset. The variance-to-mean ratio (also called index of dispersion), *VMR_ti _*(Eq.4), is a quantitative measure of the degree of clustering of concept annotations. A ratio that is greater than 1 indicates a clustered distribution, i.e., concept annotations are unevenly distributed in the subset *docs_ti_*; less than 1 indicates an evenly dispersed distribution, i.e., concept annotations are evenly distributed in the subset *docs_ti_*; equal to 1, a random distribution; and, equal to 0, indicates a constant distribution, i.e., the number of concept annotations is the same in all documents that refer to the concept. Finally, the frequency of co-annotation relates two different entities, assuming that entities that are often co-annotated are biologically engaged [67]. The frequency of co-annotation, *freq_ti,tj _*(Eq.5), of two concepts, was estimated as the number of documents in which concepts *t_i _*and *t_j _*co-occur divided by the number of documents in which the concept *t_i _*appears (*t_i _*is used as the reference concept). These interactions were illustrated using the Cytoscape biomolecular interaction viewer and analyser [67].

The statistics of the ontology assignments were employed in both process and functional analyses. The functions of the annotated gene products and the involved biological processes are reflected in their GO and MultiFun annotations and thus, processes and functions of interest were identified by finding statistically enriched terms (mainly by looking into the frequency of annotation). The frequency of assignment of ontology terms was estimated the as the fraction of documents where the assigning concepts were annotated. For example, if a GO term is assigned to one or more concepts that were annotated in 80% of the documents, than the frequency of that ontology term in the corpus is 80%. The frequency of annotation of a given term (A) by a given concept (B) was therefore estimated based on the ratio of the number of annotations of the concept (B) by the number of times that ontology term (A) was assigned by any concept associated to that term (A). Since one concept can be associated to several ontology terms, it can be considered that the under- or over-representation of an ontology term can depend on the number of annotations of the assigned concepts.

Similar assessments were taken over PSI-MI assignments towards the identification of the techniques that have contributed the most to the study of the stringent response. Apart from the systematic analysis of the set of annotations in the corpus, a retrospective analysis of annotations per decade was undertaken (i.e. frequency of annotation per decade). Such analysis aimed at looking into the evolving experimental techniques that contributed to the study of the stringent response over the decades and, in particular, evaluating the impact that the technological evolution had in the identification of molecular participants.

Finally, a set of recent documents that review the literature on the subject were manually retrieved from PubMed. Their contents were evaluated in terms of annotations of genetic components, gene products and small molecules and further compared to the annotations retrieved from the corpus.

## Competing interests

The authors declare that they have no competing interests.

## Authors' contributions

All authors participated in the preparation of the manuscript. AL and SC implemented the text mining methodology. SC performed the annotations of biological concepts in the full-text documents. AL performed the corpus analysis and ECF supervised most of the statistical analysis. IR supervised all steps of the work. All authors read and approved the final manuscript.

## Endnotes

^1^The PubMed Unique Identifiers (PMIDs) indicate which documents from the corpus supported the evidences.

## Supplementary Material

Additional file 1 **An Excel file with the list of documents analysed in this study**. Click here for file

Additional file 2 **An Excel file with some details about the corpus analysis**. Click here for file

Additional file 3 **An Excel file with the list of co-annotations between ppGpp, (p)ppGpp and ppGpp nucleotides and gene products**. Click here for file

Additional file 4 **An Excel file comparing the extent of biological concepts described in the corpus and selected reviews**. Click here for file

Additional file 5 **An Excel file with the list of annotations and statistical analysis of genetic components (DNA, RNA and genes)**. Click here for file

Additional file 6 **An Excel file with the list of annotations and statistical analysis of gene products (enzymes, transcriptional factors and other proteins)**. Click here for file

Additional file 7 **An Excel file with the list of annotations and statistical analysis of small molecules**. Click here for file

## References

[B1] Garten Y Coulet A Altman RB Recent progress in automatically extracting information from the pharmacogenomic literature Pharmacogenomics 2010 11 1467 1489 10.2217/pgs.10.136 21047206 PMC3035632

[B2] Frijters R van VM Smeets R van SR de VJ Alkema W Literature mining for the discovery of hidden connections between drugs, genes and diseases PLoS Comput Biol 2010 6 10.1371/journal.pcbi.1000943 PMC2944780 20885778

[B3] Nobata C Dobson PD Iqbal SA Mendes P Tsujii J Kell DB Mining metabolites: extracting the yeast metabolome from the literature Metabolomics 2011 7 94 101 10.1007/s11306-010-0251-6 21687783 PMC3111869

[B4] Banville DL Mining chemical and biological information from the drug literature Current Opinion in Drug Discovery & Development 2009 12 376 387 19396739

[B5] Winnenburg R Wachter T Plake C Doms A Schroeder M Facts from text: can text mining help to scale-up high-quality manual curation of gene products with ontologies Briefings in Bioinformatics 2008 9 466 478 10.1093/bib/bbn043 19060303

[B6] He X Li YN Khetani R Sanders B Lu Y Ling X BSQA: integrated text mining using entity relation semantics extracted from biological literature of insects Nucl Acids Res 2010 38 W175 W181 10.1093/nar/gkq544 20576702 PMC2896161

[B7] Waagmeester A Pezik P Coort S Tourniaire F Evelo C Rebholz-Schuhmann D Pathway enrichment based on text mining and its validation on carotenoid and vitamin A metabolism Omics-A Journal of Integrative Biology 2009 13 367 379 10.1089/omi.2009.0029 19715393

[B8] Carneiro S Villas-Boas SG Ferreira EC Rocha I A systematic modeling approach to elucidate the triggering of the stringent response in recombinant *E. coli *systems Advances in Intelligent and Soft Computing 2011 Springer Berlin/Heidelberg

[B9] Jain V Kumar M Chatterji D ppGpp: Stringent response and survival Journal of Microbiology 2006 44 1 10 16554711

[B10] Battesti A Bouveret E Bacteria possessing two RelA/SpoT-like proteins have evolved a specific stringent response involving the Acyl Carrier Protein-SpoT interaction Journal of Bacteriology 2009 191 616 624 10.1128/JB.01195-08 18996989 PMC2620808

[B11] Srivatsan A Wang JD Control of bacterial transcription, translation and replication by (p)ppGpp Current Opinion in Microbiology 2008 11 100 105 10.1016/j.mib.2008.02.001 18359660

[B12] Chang DE Smalley DJ Conway T Gene expression profiling of *Escherichia coli *growth transitions: an expanded stringent response model Molecular Microbiology 2002 45 289 306 10.1046/j.1365-2958.2002.03001.x 12123445

[B13] Murray KD Bremer H Control of *spo*T-dependent ppGpp synthesis and degradation in *Escherichia coli* Journal of Molecular Biology 1996 259 41 57 10.1006/jmbi.1996.0300 8648647

[B14] Xiao H Kalman M Ikehara K Zemel S Glaser G Cashel M Residual guanosine 3',5'-bispyrophosphate synthetic activity of *rel*A null mutants can be eliminated by *spo*T null mutations Journal of Biological Chemistry 1991 266 5980 5990 2005134

[B15] Roberts JW Promoter-specific control of *E. coli *RNA polymerase by ppGpp and a general transcription factor Genes & Development 2009 23 143 146 10.1101/gad.1770509 19171778

[B16] Chatterji D Ojha AK Revisiting the stringent response, ppGpp and starvation signaling Current Opinion in Microbiology 2001 4 160 165 10.1016/S1369-5274(00)00182-X 11282471

[B17] Magnusson LU Farewell A Nystrom T ppGpp: a global regulator in *Escherichia coli* Trends in Microbiology 2005 13 236 242 10.1016/j.tim.2005.03.008 15866041

[B18] Baracchini E Bremer H Stringent and growth control of ribosomal RNA synthesis in *Escherichia coli *are both mediated by ppGpp Journal of Biological Chemistry 1988 263 2597 2602 2449428

[B19] Torok I Kari C Accumulation of ppGpp in a *rel*A mutant of *Escherichia coli *during amino acid starvation Journal of Biological Chemistry 1980 255 3838 3840 6768741

[B20] Johnson GS Adler CR Collins JJ Court D Role of the *spo*T gene product and manganese ion in the metabolism of guanosine 5'-diphosphate 3'-diphosphate in *Escherichia coli* Journal of Biological Chemistry 1979 254 5483 5487 376509

[B21] Chatterji D Fujita N Ishihama A The mediator for stringent control, ppGpp, binds to the beta-subunit of *Escherichia coli *RNA polymerase Genes to Cells 1998 3 279 287 10.1046/j.1365-2443.1998.00190.x 9685179

[B22] Artsimovitch I Patlan V Sekine SI Vassylyeva MN Hosaka T Ochi K Structural basis for transcription regulation by alarmone ppGpp Cell 2004 117 299 310 10.1016/S0092-8674(04)00401-5 15109491

[B23] Paul BJ Barker MM Ross W Schneider DA Webb C Foster JW DksA: A critical component of the transcription initiation machinery that potentiates the regulation of rRNA promoters by ppGpp and the initiating NTP Cell 2004 118 311 322 10.1016/j.cell.2004.07.009 15294157

[B24] Potrykus K Cashel M (p)ppGpp: Still Magical? Annual Review of Microbiology 2008 62 35 51 10.1146/annurev.micro.62.081307.162903 18454629

[B25] Wu J Xie J Magic spot: (p) ppGpp Journal of Cellular Physiology 2009 220 297 302 10.1002/jcp.21797 19391118

[B26] Kanjee U Gutsche I Alexopoulos E Zhao BY El Bakkouri M Thibault G Linkage between the bacterial acid stress and stringent responses: the structure of the inducible lysine decarboxylase Embo Journal 2011 30 931 944 10.1038/emboj.2011.5 21278708 PMC3049219

[B27] Traxler MF Zacharia VM Marquardt S Summers SM Nguyen HT Stark SE Discretely calibrated regulatory loops controlled by ppGpp partition gene induction across the 'feast to famine' gradient in *Escherichia coli* Molecular Microbiology 2011 79 830 845 10.1111/j.1365-2958.2010.07498.x 21299642 PMC3073637

[B28] Keseler IM Bonavides-Martinez C Collado-Vides J Gama-Castro S Gunsalus RP Johnson DA EcoCyc: a comprehensive view of *Escherichia coli *biology Nucl Acids Res 2009 37 D464 D470 10.1093/nar/gkn751 18974181 PMC2686493

[B29] Hermjakob H Montecchi-Palazzi L Bader G Wojcik R Salwinski L Ceol A The HUPOPSI's Molecular Interaction format - a community standard for the representation of protein interaction data Nature Biotechnology 2004 22 177 183 10.1038/nbt926 14755292

[B30] Serres MH Riley M MultiFun, a multifunctional classification scheme for *Escherichia coli *K-12 gene products Microbial & Comparative Genomics 2000 5 205 222 11471834 10.1089/omi.1.2000.5.205

[B31] Ashburner M Ball CA Blake JA Botstein D Butler H Cherry JM Gene Ontology: tool for the unification of biology Nature Genetics 2000 25 25 29 10.1038/75556 10802651 PMC3037419

[B32] Wren JD Bekeredjian R Stewart JA Shohet RV Garner HR Knowledge discovery by automated identification and ranking of implicit relationships Bioinformatics 2004 20 389 398 10.1093/bioinformatics/btg421 14960466

[B33] Walker KA Atkins CL Osuna R Functional determinants of the *Escherichia coli fis *promoter: roles of -35, -10, and transcription initiation regions in the response to stringent control and growth phase-dependent regulation Journal of Bacteriology 1999 181 1269 1280 9973355 10.1128/jb.181.4.1269-1280.1999 PMC93506

[B34] Ross W Thompson JF Newlands JT Gourse RL *E.coli *Fis protein activates ribosomal RNA transcription *in vitro *and *in vivo* EMBO J 1990 9 3733 3742 2209559 10.1002/j.1460-2075.1990.tb07586.x PMC552129

[B35] Shiba T Tsutsumi K Yano H Ihara Y Kameda A Tanaka K Inorganic polyphosphate and the induction of *rpo*S expression Proceedings of the National Academy of Sciences of the United States of America 1997 94 11210 11215 10.1073/pnas.94.21.11210 9326588 PMC23418

[B36] Condon C Squires C Squires CL Control of ribosomal RNA transcription in *Escherichia coli* Microbiological Reviews 1995 59 623 & 8531889 10.1128/mr.59.4.623-645.1995 PMC239391

[B37] Hara A Sy J Guanosine 5'-triphosphate, 3'-diphosphate 5'-phosphohydrolase. Purification and substrate specificity Journal of Biological Chemistry 1983 258 1678 1683 6130093

[B38] Manganelli R Polyphosphate and stress response in mycobacteria Molecular Microbiology 2007 65 258 260 10.1111/j.1365-2958.2007.05819.x 17590232

[B39] Jenvert RM Schiavone LH The flexible N-terminal domain of ribosomal protein L11 from *Escherichia coli *is necessary for the activation of stringent factor Journal of Molecular Biology 2007 365 764 772 10.1016/j.jmb.2006.10.065 17095013

[B40] Wendrich TM Blaha G Wilson DN Marahiel MA Nierhaus KH Dissection of the mechanism for the stringent factor RelA Molecular Cell 2002 10 779 788 10.1016/S1097-2765(02)00656-1 12419222

[B41] Rao NN Liu SJ Kornberg A Inorganic polyphosphate in *Escherichia coli*: the phosphate regulon and the stringent response Journal of Bacteriology 1998 180 2186 2193 9555903 10.1128/jb.180.8.2186-2193.1998 PMC107147

[B42] Vinella D Albrecht C Cashel M D'Ari R Iron limitation induces SpoT-dependent accumulation of ppGpp in *Escherichia coli* Molecular Microbiology 2005 56 958 970 10.1111/j.1365-2958.2005.04601.x 15853883

[B43] Johansson J Balsalobre C Wang SY Urbonaviciene J Jin DJ Sonden B Nucleoid proteins stimulate stringently controlled bacterial promoters: A link between the cAMP-CRP and the (p)ppGpp regulons in *Escherichia coli* Cell 2000 102 475 485 10.1016/S0092-8674(00)00052-0 10966109

[B44] Chiaramello AE Zyskind JW Coupling of DNA replication to growth rate in *Escherichia coli*: a possible role for guanosine tetraphosphate Journal of Bacteriology 1990 172 2013 2019 1690706 10.1128/jb.172.4.2013-2019.1990 PMC208699

[B45] Beissbarth T Speed TP GOstat: find statistically overrepresented Gene Ontologies within a group of genes Bioinformatics 2004 20 1464 1465 10.1093/bioinformatics/bth088 14962934

[B46] Martin D Brun C Remy E Mouren P Thieffry D Jacq B GOToolBox: functional analysis of gene datasets based on Gene Ontology Genome Biology 2004 5 10.1186/gb-2004-5-12-r101 PMC545796 15575967

[B47] Ananiadou S Pyysalo S Tsujii J Kell DB Event extraction for systems biology by text mining the literature Trends in Biotechnology 2010 28 381 390 10.1016/j.tibtech.2010.04.005 20570001

[B48] Hakenberg J Schmeier S Kowald A Klipp E Leser U Finding kinetic parameters using text mining Omics-A Journal of Integrative Biology 2004 8 131 152 10.1089/1536231041388366 15268772

[B49] Jensen LJ Saric J Bork P Literature mining for the biologist: from information retrieval to biological discovery Nature Reviews Genetics 2006 7 119 129 10.1038/nrg1768 16418747

[B50] Muller HM Kenny EE Sternberg PW Textpresso: An ontology-based information retrieval and extraction system for biological literature Plos Biology 2004 2 1984 1998 10.1371/journal.pbio.0020309 PMC517822 15383839

[B51] Cashel M Kalbache B Control of ribonucleic acid synthesis in *Escherichia coli *5. Characterization of a nucleotide associated with stringent response Journal of Biological Chemistry 1970 245 2309 4315151

[B52] Lagosky PA Chang FN Influence of amino acid starvation on guanosine 5'-diphosphate 3'-diphosphate basal-level synthesis in *Escherichia coli* Journal of Bacteriology 1980 144 499 508 6159345 10.1128/jb.144.2.499-508.1980 PMC294696

[B53] Liang ST Xu YC Dennis P Bremer H mRNA composition and control of bacterial gene expression Journal of Bacteriology 2000 182 3037 3044 10.1128/JB.182.11.3037-3044.2000 10809680 PMC94487

[B54] Irr JD Control of nucleotide metabolism and ribosomal ribonucleic acid synthesis during nitrogen starvation of *Escherichia coli* Journal of Bacteriology 1972 110 554 561 4553835 10.1128/jb.110.2.554-561.1972 PMC247448

[B55] Jishage M Kvint K Shingler V Nystrom T Regulation of or factor competition by the alarmone ppGpp Genes & Development 2002 16 1260 1270 10.1101/gad.227902 12023304 PMC186289

[B56] Zhou YN Jin DJ The *rpo*B mutants destabilizing initiation complexes at stringently controlled promoters behave like "stringent" RNA polymerases in *Escherichia coli* Proceedings of the National Academy of Sciences of the United States of America 1998 95 2908 2913 10.1073/pnas.95.6.2908 9501189 PMC19668

[B57] Selby CP Sancar A Mechanisms of transcription-repair coupling and mutation frequency decline Microbiological Reviews 1994 58 317 329 7968917 10.1128/mr.58.3.317-329.1994 PMC372971

[B58] Trautinger BW Jaktaji RP Rusakova E Lloyd RG RNA polymerase modulators and DNA repair activities resolve conflicts between DNA replication and transcription Molecular Cell 2005 19 247 258 10.1016/j.molcel.2005.06.004 16039593

[B59] Pingoud A Block W The elongation factor Tu guanosine tetraphosphate complex Eur J Biochem 1981 116 631 634 10.1111/j.1432-1033.1981.tb05382.x 7021151

[B60] Pingoud A Gast FU Block W Peters F The elongation factor Tu from *Escherichia coli*, aminoacyl-tRNA, and guanosine tetraphosphate form a ternary complex which is bound by programmed ribosomes J Biol Chem 1983 258 14200 14205 6358217

[B61] Yang X Ishiguro EE Involvement of the N terminus of ribosomal protein L11 in regulation of the RelA protein of *Escherichia coli* Journal of Bacteriology 2001 183 6532 6537 10.1128/JB.183.22.6532-6537.2001 11673421 PMC95482

[B62] Nakanishi N Abe H Ogura Y Hayashi T Tashiro K Kuhara S ppGpp with DksA controls gene expression in the locus of enterocyte effacement (LEE) pathogenicity island of enterohaemorrhagic *Escherichia coli *through activation of two virulence regulatory genes Mol Microbiol 2006 61 194 205 10.1111/j.1365-2958.2006.05217.x 16824105

[B63] Bichara M Pinet I Lambert LB Fuchs RPP RecA-mediated excision repair: a novel mechanism for repairing DNA lesions at sites of arrested DNA synthesis Molecular Microbiology 2007 65 218 229 10.1111/j.1365-2958.2007.05790.x 17581130

[B64] Durfee T Hansen AM Zhi H Blattner FR Jin DJ Transcription profiling of the stringent response in *Escherichia coli* Journal of Bacteriology 2008 190 1084 1096 10.1128/JB.01092-07 18039766 PMC2223561

[B65] Chatterji D Ogawa Y Shimada T Ishihama A The role of the omega subunit of RNA polymerase in expression of the *rel*A gene in *Escherichia coli* Fems Microbiology Letters 2007 267 51 55 10.1111/j.1574-6968.2006.00532.x 17233676

[B66] Lourenco A Carreira R Carneiro S Maia P Glez-Pena D Fdez-Riverola F @Note: a workbench for biomedical text mining J Biomed Inform 2009 42 710 720 10.1016/j.jbi.2009.04.002 19393341

[B67] Shannon P Markiel A Ozier O Baliga NS Wang JT Ramage D Cytoscape: a software environment for integrated models of biomolecular interaction networks Genome Research 2003 13 2498 2504 10.1101/gr.1239303 14597658 PMC403769

[B68] Jones PG Cashel M Glaser G Neidhardt FC Function of a relaxed-like state following temperature downshifts in *Escherichia coli* Journal of Bacteriology 1992 174 3903 3914 1597413 10.1128/jb.174.12.3903-3914.1992 PMC206098

[B69] Mogk A Deuerling E Vorderwulbecke S Vierling E Bukau B Small heat shock proteins, ClpB and the DnaK system form a functional triade in reversing protein aggregation Molecular Microbiology 2003 50 585 595 10.1046/j.1365-2958.2003.03710.x 14617181

[B70] Shinagawa H Makino K Amemura M Kimura S Iwasaki H Nakata A Structure and regulation of the *Escherichia coli ruv *operon involved in DNA repair and recombination Journal of Bacteriology 1988 170 4322 4329 2842314 10.1128/jb.170.9.4322-4329.1988 PMC211445

[B71] Sabnis NA Yang H Romeo T Pleiotropic regulation of central carbohydrate metabolism in *Escherichia coli *via the gene *csr*A Journal of Biological Chemistry 1995 270 29096 29104 10.1074/jbc.270.49.29096 7493933

[B72] Pernestig AK Melefors O Georgellis D Identification of UvrY as the cognate response regulator for the BarA sensor kinase in *Escherichia coli* Journal of Biological Chemistry 2001 276 225 231 11022030 10.1074/jbc.M001550200

[B73] Dubey AK Baker CS Suzuki K Jones AD Pandit P Romeo T CsrA regulates translation of the *Escherichia coli *carbon starvation gene, *cst*A, by blocking ribosome access to the *cst*A transcript Journal of Bacteriology 2003 185 4450 4460 10.1128/JB.185.15.4450-4460.2003 12867454 PMC165747

[B74] Yamanaka K Inouye M Growth-phase-dependent expression of *csp*D, encoding a member of the CspA family in *Escherichia coli* Journal of Bacteriology 1997 179 5126 5130 9260955 10.1128/jb.179.16.5126-5130.1997 PMC179371

[B75] Podkovyrov SM Larson TJ Identification of promoter and stringent regulation of transcription of the *fab*H, *fab*D and *fab*G genes encoding fatty acid biosynthetic enzymes of *Escherichia coli* Nucl Acids Res 1996 24 1747 1752 10.1093/nar/24.9.1747 8649995 PMC145835

[B76] Peterson CN Mandel MJ Silhavy TJ *Escherichia coli *starvation diets: essential nutrients weigh in distinctly Journal of Bacteriology 2005 187 7549 7553 10.1128/JB.187.22.7549-7553.2005 16267278 PMC1280323

[B77] Gong L Takayama K Kjelleberg S Role of *spo*T-dependent ppGpp accumulation in the survival of light-exposed starved bacteria Microbiology 2002 148 559 570 11832519 10.1099/00221287-148-2-559

[B78] Almiron M Link AJ Furlong D Kolter R A novel DNA-binding protein with regulatory and protective roles in starved *Escherichia coli* Genes Dev 1992 6 2646 2654 10.1101/gad.6.12b.2646 1340475

[B79] Aizenman E Engelberg-Kulka H Glaser G An *Escherichia coli *chromosomal "addiction module" regulated by guanosine 3',5'-bispyrophosphate: a model for programmed bacterial cell death Proceedings of the National Academy of Sciences of the United States of America 1996 93 6059 6063 10.1073/pnas.93.12.6059 8650219 PMC39188

[B80] Gross M Marianovsky I Glaser G MazG -- a regulator of programmed cell death in *Escherichia coli* Mol Microbiol 2006 59 590 601 10.1111/j.1365-2958.2005.04956.x 16390452

